# Tool bending in New Caledonian crows

**DOI:** 10.1098/rsos.160439

**Published:** 2016-08-10

**Authors:** Christian Rutz, Shoko Sugasawa, Jessica E. M. van der Wal, Barbara C. Klump, James J. H. St Clair

**Affiliations:** Centre for Biological Diversity, School of Biology, University of St Andrews, St Andrews KY16 9TH, UK

**Keywords:** comparative cognition, *Corvus moneduloides*, innovation, insight, intelligence, tool use

## Abstract

‘Betty’ the New Caledonian crow astonished the world when she ‘spontaneously’ bent straight pieces of garden wire into hooked foraging tools. Recent field experiments have revealed that tool bending is part of the species' natural behavioural repertoire, providing important context for interpreting Betty's iconic wire-bending feat. More generally, this discovery provides a compelling illustration of how natural history observations can inform laboratory-based research into the cognitive capacities of non-human animals.

In 2002, a captive New Caledonian crow *Corvus moneduloides*—named Betty—bent straight pieces of garden wire into hooked tools, which she subsequently used for lifting a small food-baited bucket from a plastic well (figure 1*a*; electronic supplementary material, movie S1, *Scene* 1; [1]). Although it was known at the time that these tropical corvids manufacture hooked foraging tools from forked twigs in the wild [2], Betty's wire-bending method appeared to be a spontaneous, innovative solution to a novel problem. The paper that described these observations shook the field of comparative cognition, and quickly became a textbook example of ‘animal intelligence’ (for selected references, see [3]). In an unexpected twist, we have recently discovered that tool bending is part of New Caledonian crows' natural behavioural repertoire [4], providing crucial context for this iconic experiment.

**Figure 1.**
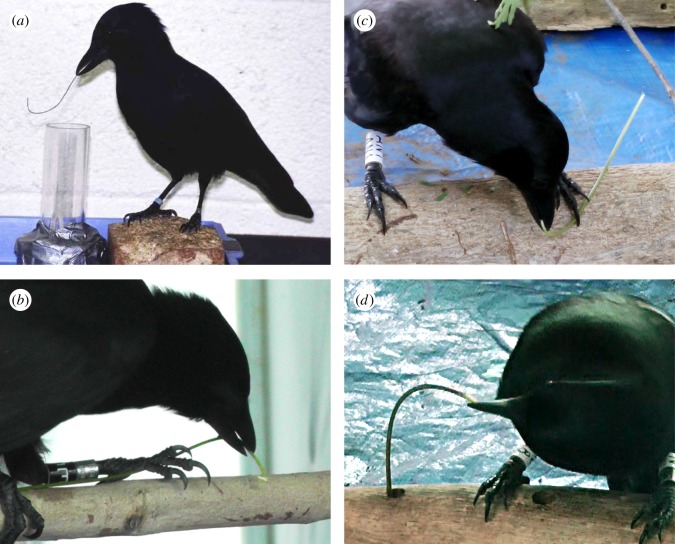
Tool-bending behaviour in New Caledonian crows. (*a*) ‘Betty’ about to use a bent piece of garden wire to lift a baited container from a vertical tube (from [1]; reproduced with permission from AAAS). (*b*–*d*) Temporarily captive crows using a range of techniques for bending the shaft of hooked stick tools during natural tool manufacture (from [4,7]).

New Caledonian crows are the only non-human animals known to craft hooks in the wild [5]. This remarkable discovery was based on fleeting glimpses of four cases of hook-tool making [2], and it was only years later that researchers managed to attract two wild crows to baited feeding tables, providing close-up views of 10 manufacture episodes [6]. These observations suggested that hook-tool making follows a consistent pattern: crows detached side branches from vegetation, and subsequently ‘crafted’ a neat terminal hook from the joint that had originally connected the branch to the main stem—a process that involved dexterous removal of small wood pieces using the bill, but no bending. Following manufacture, birds used these tools to extract meat from an experimental task.

Over the last four years, we have conducted a suite of experiments to investigate New Caledonian crows' natural hook-making techniques, which provided fresh clues about the possible origins of Betty's behaviour. In a key experiment, 18 wild-caught crows produced a total of 85 hooked stick tools under controlled conditions in field aviaries, to extract small pieces of food from holes drilled into wooden logs [4]. Unexpectedly, after detaching a side branch and (usually) some processing of the hook, just as described earlier [6] (see above), 10 of our subjects deliberately bent their tools (during some or all of their manufactures), apparently attempting to induce extra curvature to the working end of the tool shaft (figure 1*b–d*; electronic supplementary material, movie S1, *Scenes* 3–6; [4])—a behaviour that we have since confirmed in the wild (electronic supplementary material, movie S1, *Scene* 10). Importantly, results of a separate experiment strongly suggested that tool curvature has functional significance [7]: when presented with a non-hooked stick tool that was straight at one end and slightly curved at the other, eight of eight crows immediately used the curved end of the tool to probe for food; moreover, in a different treatment, five of these subjects bent supplied, artificially straight-shafted hooked stick tools (made from natural materials) before deployment (electronic supplementary material, movie S1, *Scenes* 7–9; [7]). Modifying shaft curvature may improve tool function, for example by centring the terminal hook in the birds' visual field [3,8], but it is not necessary for hook production [4].

Birds in this particular study population prefer to make their hooked stick tools from the perennial shrub *Desmanthus virgatus* [9]. These plants have pliable side branches that, although less flexible and more elastic than garden wire, can be permanently deformed. Reshaping is *not* an inevitable consequence of material properties and routine handling, but requires deliberate flexing of the material well beyond its final desired form, which would explain why crows often bend stems so vigorously during tool manufacture (electronic supplementary material, movie S1, *Scenes* 3–9; [2,3]). When applied to more pliant materials, like metal wires or strips, such forceful actions produce pronounced bends, as indeed observed in the experiment with Betty (see fig. 1*b* in [1]).

Betty's actions [1,10] were highly reminiscent of the tool bending exhibited by wild crows in our recent field studies [4,7] (electronic supplementary material, movie S1). So far, we have observed three distinct bending techniques [4]: most birds trapped sticks underfoot before bending the tool shaft by pulling and/or controlled rotation of the bill (45 times by 10 subjects), but one also pushed tools against hard surfaces to flex the functional end (8 times), and another wedged them upright into holes before pulling the shaft sideways (3 times), just as Betty had done [1,10]. Our findings therefore suggest that Betty, who had been wild-caught as a young bird, may well have expressed behaviours during test trials that were an existing part of her natural repertoire, rather than innovative solutions to an unfamiliar problem.

In a follow-up study, Betty developed, over multiple trials, different bending techniques for a novel material (aluminium strips), adding curvature to straight tools, and reducing it in U-shaped ones ([10,11]; for further discussion, see [3]). Plasticity in technique has not yet been formally investigated with crows handling natural plant materials, but two individuals have been observed to employ multiple bending techniques with *Desmanthus* stems (electronic supplementary material, movie S1, *Scenes* 5,6; [4]), and subjects often make deliberate tool-shaft adjustments during foraging (electronic supplementary material, movie S1, *Scenes* 3,6; [4]), suggesting that bending behaviour is indeed sensitive to task requirements. Betty had also used (non- or minimally pliable) sticks, bamboo skewers and thin dowelling as tools in a range of tool-selectivity experiments, for which no (attempted) bending was reported [12–14]. Our field studies not only demonstrate within- and between-bird variation in bending behaviour [4], but also indicate some context dependence: in certain foraging situations (e.g. ‘larva fishing’, [15]), and when using certain tool types/materials (non-hooked stick tools in recent experiments), bending seems comparatively rare. We are currently investigating these issues in dedicated experiments.

Although Betty sadly died in 2005, subsequent experiments documented bending behaviour for another eight permanently captive wild-caught New Caledonian crows [16]. These experiments have not yet been published in the peer-reviewed literature, but based on preliminary analyses presented in an MSc thesis [16], we conclude that they do not provide convincing evidence for insightful behaviour. Not all tested subjects were found to bend their tools, exactly as observed in our study [4], and those that did exhibit the behaviour collectively employed all three natural bending techniques described above (unfortunately, we were unable to ascertain exact sample sizes from the information provided in [16]). Taken together, these studies show that the behavioural actions crows use for shaping hooks from flexible metal materials closely match those expressed by wild birds for routine bending of their hooked stick tools (two crows have been observed to fold, and sometimes accidentally bend, a different tool type—rectangular sections cut from the barbed edges of *Pandanus* spp. leaves; [17]).

Our observations do not rule out that Betty had some ‘causal understanding’ of task requirements or was capable of ‘inventing’ novel behavioural solutions, as discussed at length elsewhere ([3]; for a general review of corvid cognition, see [18]), but they do provide an alternative explanation: she may have simply followed pre-existing tool-manipulation routines. While the tool-related cognition of New Caledonian crows is undoubtedly far from ‘simple’, involving interactions between genetic predispositions [19], and individual and social learning [20,21], our work suggests that Betty's performance can potentially be explained without invoking exceptional cognitive processes (see also [22,23]). At the very least, this new evidence suggests that the most compelling case to date of spontaneous hook shaping from supplied wire pieces comes from a different corvid species, the rook *C. frugilegus* [24], for which we can more confidently exclude a predisposition to bend tools. Rooks have not been observed to use foraging tools in the wild, despite extensive research and their commensal presence in human settlements [25], although future work needs to establish whether they bend materials as part of nest construction.

The extraordinary richness of animal behaviour implies that it is unrealistic to chart a species' full natural behavioural repertoire before adopting it as a laboratory subject. What can be done, however, is to consider whether an animal's performance *might* result from a predisposition (or inability) to express behaviours of interest, and to control experimentally for this possibility. This point is perennially raised in the comparative cognition literature [26], but can be surprisingly difficult to address (for an excellent example of how accounting for species-typical behavioural predispositions can affect task performance, see [27]). The authors of the original Betty paper were appropriately cautious with their claims [1], and certainly could not have known about wild New Caledonian crows' tool-bending techniques given the paucity of field observations at the time (for a review, see [28]). But, we suggest that the addition of a simple control treatment—a task in which it was *not* necessary to bend the wire—would have permitted stronger inference about Betty's cognitive capabilities. Documenting natural animal behaviour can be logistically challenging and time-consuming [2,29], but remains immensely important in guiding experimental research in cognition, and—as shown here—in interpreting its results [30–32].
